# Production of ρ-Hydroxyacetophenone by Engineered *Escherichia coli* Heterologously Expressing 1-(4-Hydroxyphenyl)-Ethanol Dehydrogenase

**DOI:** 10.4014/jmb.2310.10019

**Published:** 2023-12-08

**Authors:** Wenmei Wu, Xiwei Yuan, Xin Gao, Chaoyang Tan, Shunxiang Li, Dehong Xu

**Affiliations:** 1 Biological Engineering Laboratory, College of Pharmacy, Hunan University of Chinese Medicine, Changsha, Hunan 410208, P.R. China; 2 Hunan Engineering Technology Research Center for Bioactive Substance Discovery of Chinese Medicine, Changsha, Hunan 410208, P.R. China; 3 Hunan Province Sino-US International Joint Research Center for Therapeutic Drugs of Senile Degenerative Diseases, Changsha, Hunan 410208, P.R. China

**Keywords:** ρ-Hydroxyacetophenone, *Escherichia coli*, biotransformation, 1-(4-hydroxyphenyl)-ethanol dehydrogenase

## Abstract

ρ-Hydroxyacetophenone is an important and versatile compound that has been widely used in medicine, cosmetics, new materials, and other fields. At present, there are two ways to obtain ρ-hydroxyacetophenone. One is to extract it from plants, such as *Artemisia capillaris* Thunb and *Cynanchum otophyllum* Schneid, and the other is to synthesize it by using chemical methods. Of these two methods, the second is the main one, although it has problems, such as flammable and explosive reagents, difficult separation of by-products, and harsh reaction conditions. To solve these issues, we adopted genetic engineering in this study to construct engineered *Escherichia coli* containing *Hped* gene or *EbA309* gene. Whole-cell biotransformation was conducted under the same conditions to select the engineered *E. coli* with the higher activity. Orthogonal tests were conducted to determine the optimal biotransformation condition of the engineered *E. coli*. The results showed that the optimal condition was as follows: substrate concentration of 40 mmol/l, IPTG concentration of 0.1 mmol/l, an induction temperature of 25°C, and a transformation temperature of 35°C. Under this condition, the effects of transformation time on the ρ-hydroxyacetophenone concentration and cell growth were further studied. We found that as the transformation time extended, the ρ-hydroxyacetophenone concentration showed a gradually increasing trend. However, when the ρ-hydroxyacetophenone concentration increased to 1583.19 ± 44.34 mg/l in 24 h, cell growth was inhibited and then entered a plateau. In this research, we realized the synthesis of ρ-hydroxyacetophenone by biotransformation, and our findings lay a preliminary foundation for further improving and developing this method.

## Introduction

Para-hydroxyacetophenone (ρ-HAP), commonly known as piceol, has hydroxyl and keto groups on the phenyl ring of its molecular structure, with the properties of phenol and aromatic ketone compounds. According to relevant assays, ρ-HAP is naturally present in a variety of medicinal plants, including among others [1] *Artemisia capillaris* Thunb in the Asteraceae family [2], and *Cynanchum otophyllum* Schneid in the Asclepiadaceae family [3]. ρ-HAP has a wide range of applications in several fields. First, ρ-HAP can be used for adjuvant treatment of acute and chronic icteric hepatitis, cholecystitis, and other diseases in the medicinal field [4, 5], in addition to being used as a raw material for the antipyretic drug paracetamol and the antiasthmatic drug salbutamol [6, 7]. Second, ρ-HAP can be utilized to produce fragrances in the field of cosmetics, and it also serves as a nontoxic and safe premium preservative [8, 9]. Finally, in the field of new materials, ρ-HAP is an important ingredient for some useful derivatives. For instance, 4-(4-phenylquinolin-2yl) phenol generated by condensation of ρ-HAP with 2-aminobenzophenone is used as a blue luminescent material for organic, light-emitting diodes [10].

At present, the demand for ρ-HAP on the market is great, so several chemical raw material synthesis companies are engaged in its production. Of them, the world-famous fragrance manufacturer Symrise fixes the price of ρ-HAP at 42,000 USD per ton, and Jiangsu Xinhan New Materials Co., Ltd., a local enterprise in China, sets the price of localized ρ-HAP at 10,337 USD per ton [11]. During the production of ρ-HAP, all of these enterprises adopt chemical synthesis methods, which can be divided into Fries rearrangement, Friedel-Crafts acylation, and diazotization by different principles. Both the Friedel-Crafts acylation and diazotization require explosive and flammable chemical reagents, which are prone to cause safety incidents [12, 13]. Although the Fries rearrangement makes up for the drawbacks of the aforementioned methods, the reaction needs to be catalyzed by aluminum chloride at high temperature and high pressure. In addition, the byproduct ortho-hydroxyacetophenone is difficult to separate [11]. As a result, all of the chemical synthesis methods have flaws that must be addressed, or other innovative approaches must be developed as an alternative.

**Fig. 1:**
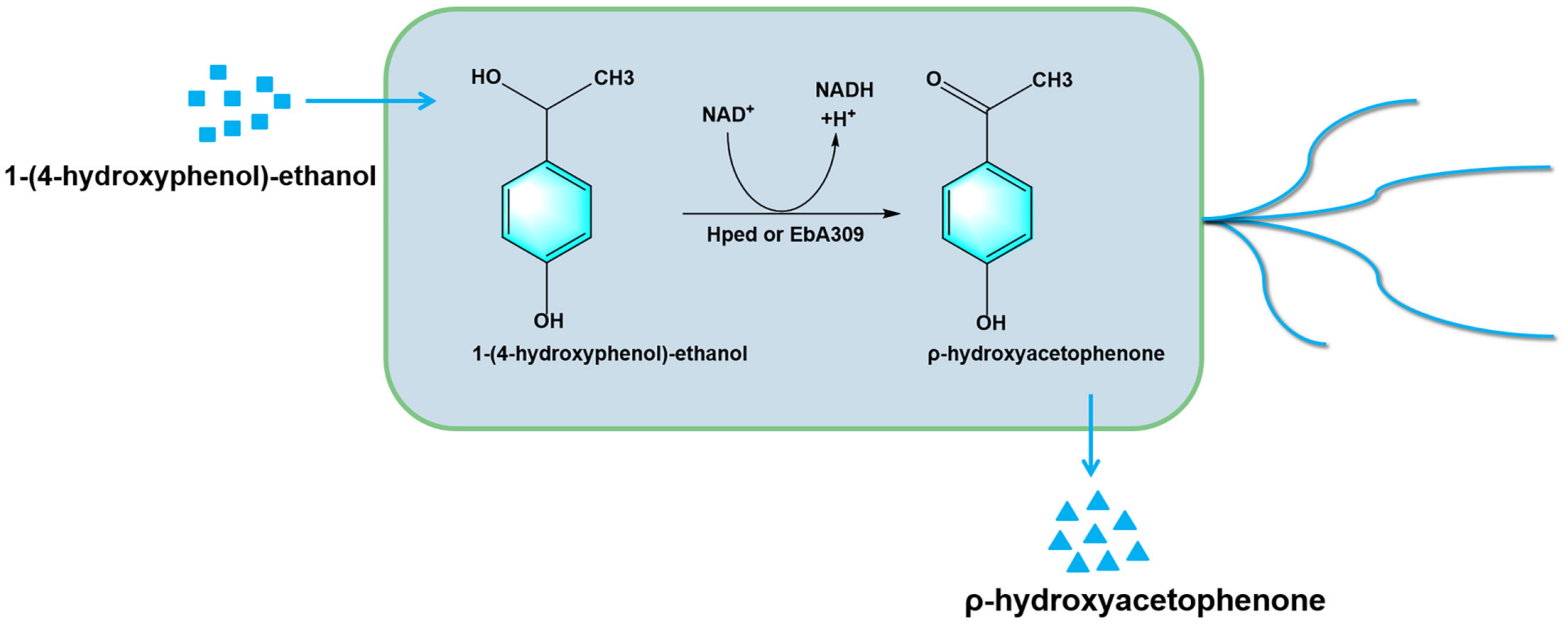
Schematic diagram of ρ-HAP biosynthesis by *E. coli* using 1-(4-hydroxybenzene)-ethanol.

In this study, two genes (*Hped* and *EbA309*) encoding isoenzymes of 1-(4-hydroxyphenol)-ethanol dehydrogenase from *Aromatoleum aromaticum* strain EbN1 were introduced into *E. coli* by genetic engineering, and then 1-(4-hydroxyphenol)-ethanol was transformed into ρ-HAP by the constructed recombinant strain (Fig. 1). The one with the higher transformation activity was used to further explore the optimal transformation condition, so as to lay a preliminary foundation for the application of the new method of ρ-HAP biosynthesis as an alternative to the old chemical synthesis method.

## Materials and Methods

### Chemicals, Media, Strains, and Vectors

*Pfu* DNA polymerase, 200 bp DNA ladder, an agarose gel DNA recovery kit, pre-stained protein marker, and a plasmid extraction kit were purchased from Tiangen (China). Restriction endonucleases (NcoI and XhoI) and T4 DNA ligase were purchased from Thermo Fisher Scientific (USA). A SDS-PAGE kit and isopropyl-β-D-thiogalactoside (IPTG) were purchased from Sangon Biotech (China). Kanamycin was purchased from Bomei Biotechnology (China). Luria-Bertani (LB) medium (5 g/l yeast extract, 10 g/l tryptone, and 10 g/l NaCl) was used for plasmid propagation, seed preparation, and protein induction expression. M9Y medium (17.1 g/l Na_2_HPO_4_·12H_2_O, 3g/l KH_2_PO_4_, 0.5 g/l NaCl, 1 g/l NH_4_Cl, 2 g/l glucose, 1 g/l yeast extract, 0.12 g/l MgSO4, 0.0111 g/l CaCl_2_) with 1-(4-hydroxyphenol)-ethanol (0.4 mM, 4 mM, and 40 mM) was used for biotransformation. The information about the strains and vectors used in this study is shown in Table 1.

### Construction of Engineered *E. coli*

The nucleic acid sequences of *Hped* and *EbA309* genes were obtained from the genome of *Aromatoleum aromaticum* strain EbN1 (NC_006513.1), and synthesized by Sangon Biotech (China) after codon optimization. The synthesized *Hped* and *EbA309* genes were employed as templates for PCR cloning, in which P-*Hped*-F and P-*EbA309*-F were used as upstream expression primers (Table 2), and P-*Hped*-R and P-*EbA309*-R as downstream expression primers (Table 2). The PCR reaction system (50 μl) consisted of 1 μl of template, 5 μl of 10× PCR buffer, 1 μl of 10 mmol/l dNTP mix, 2 μl of 10 mmol/l F primers, 2 μl of 10 mmol/l R primers, 0.5 μl of *pfu* DNA polymerase, and 38.5 μl of nuclease-free water. The PCR reaction conditions were as follows: initial denaturation at 94°C for 3 min, 35 cycles (30 s at 94°C, 30 s at 55°C, 1 min at 72°C), and final extension at 72°C for 5 min. The amplified fragments were recovered using an agarose gel DNA recovery kit. The recovered fragments and the pET-28a expression vector were then simultaneously digested by NcoI and XhoI. Finally, the two digested fragments were ligated separately to the pET-28a expression vector by T_4_ DNA ligase. The heat shock method was used to transform the constructed pET-28a-*Hped* and pET-28a-*EbA309* plasmids into *E. coli* BL21 (DE3) competent cells. The LB solid plate containing 100 μg/l kanamycin was used to select resistant recombinant *E. coli*. The one containing pET-28a-*Hped* plasmid was named S1, and the other containing pET-28a-*EbA309* plasmid was designated as S2. With the same transformation and screening method, pET-28a vector was introduced into *E. coli* BL21(DE3) competent cells to obtain control strain S0.

### Identification of Gene Expression in Engineered *E. coli*

The S0, S1 and S2 strains were inoculated separately into an LB liquid medium (5 ml) with 100 μg/l kanamycin, which was shaken overnight at 37°C and 225 r/min. The next day, the bacterial liquids were inoculated into a fresh LB liquid medium (50 ml) at a ratio of 1:50, and then cultured at 37°C and 225 r/min in a shaking flask. When the optical density at 600 nm (OD_600_) was 0.6, induction culture was conducted at 25°C and 225 r/min after the inducer (IPTG) was added to the bacterial liquids to a final concentration of 0.5 mmol/l. After 16 h of induction, 1 ml of bacterial liquids was taken from S0, S1, and S2, respectively, and used in SDS-PAGE electrophoresis experiments to identify the expression of *Hped* and *EbA309* genes.

### Comparison of Biotransformation Activity among Engineered *E. coli* Strains

After the identification of protein expression, the remaining bacterial liquids were centrifuged at 5,000 r/min for 10 min. The harvested cells were resuspended in an M9Y medium (50 ml) with 4 mmol/l (552.64 mg/l) 1-(4-hydroxyphenol)-ethanol to OD_600_=1, followed by biotransformation at 35°C and 225 r/min for 72 h. During biotransformation, 5 ml of fermentation broth was sampled from the M9Y medium every 12 h and centrifuged at 3,000 r/min for 15 min. The supernatant was collected for the detection and comparison of ρ-HAP titers.

### Exploration of the Optimal Condition for Biotransformation of Engineered *E. coli*

According to the factors and levels designed in Table 3, biotransformation orthogonal tests were conducted on the engineered *E. coli* strain with the higher transformation activity. After biotransformation for 72 h, 5 ml samples were taken from each group in the orthogonal tests to measure the ρ-HAP concentration. Based on the results of the orthogonal tests, the optimal level corresponding to each factor was then selected to analyze the biotransformation time. Finally, 5 ml samples were taken at 24, 48, 72, 96, and 120 h to explore the effect of transformation time on the ρ-HAP concentration and cell growth.

### Sample Treatment and Product Detection

The collected samples were centrifuged at 3,000 r/min for 15 min, and the supernatant was taken for freeze-drying. The freeze-dried product was redissolved with 2 ml methanol, and filtered by a 0.22-μm filter. Following that, 20 μl filtrate was taken for qualitative and quantitative analysis by high-performance liquid chromatography (HPLC, Shimadzu, Japan) equipped with a UV detector and a C18 column (4.6 × 250 mm^2^, 5 μm, ZORBAX SB, Agilent, USA). The molecular weight of the target products in the filtrate was analyzed by a high-resolution mass spectrometer (Xevo G2-XS QTof, Waters, USA).

For HPLC analysis, the mobile phase consisted of 25% methanol and 75% water. The column temperature was kept at 40°C, and the constant flow rate was 1 ml/min. The target products were detected at 276 nm.

For mass spectrometry, the detection parameters were set as follows: positive ion mode; drying gas flow rate of 6 l/min; nebulizer pressure of 40 Psig; atomization gas temperature at 325°C; sheath gas temperature at 350°C; sheath gas flow rate of 12 l/min; capillary voltage of 4000 V; mass spectrum acquisition range between 50 and 1000 m/z.

### Statistical Analysis

Data were collected from at least three individual experiments and expressed as mean ± SD. Data were analyzed using SPSS v19.0 statistical software, and *p* < 0.05 indicated significant difference.

## Results

### Construction and Gene Expression of Engineered *E. coli*

**Fig. 2:**
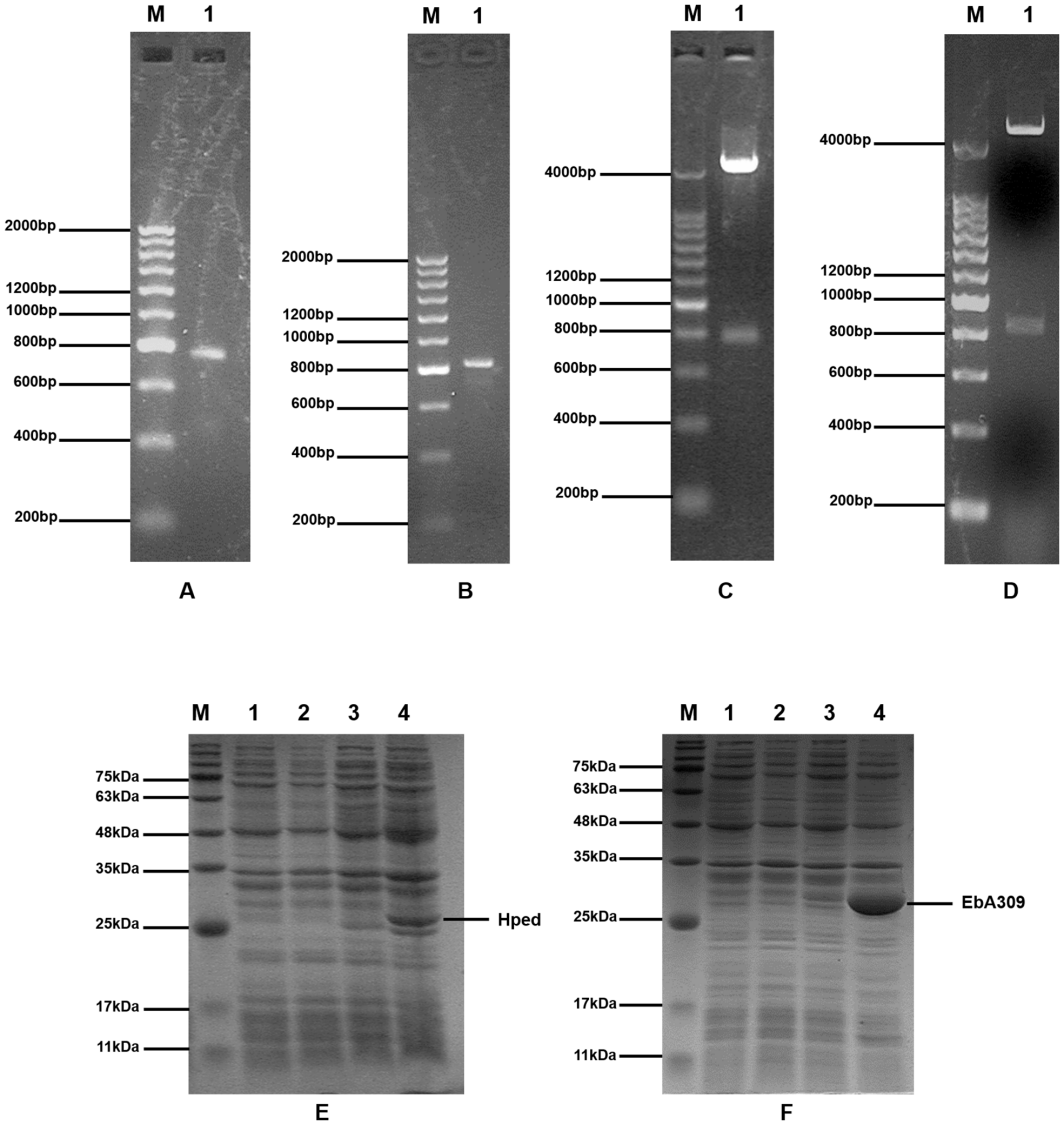
Plasmid construction and gene expression. (**A** and **B**) The PCR results of *Hped* gene and *EbA309* gene. M is DNA ladder and lane 1 is the PCR products. (**C** and **D**) Double digestion of pET-28a-*Hped* plasmid and pET-28a-*EbA309* plasmid. M is DNA Ladder and lane 1 is the double digestion products with NcoI and XhoI. (**E** and **F**) Gene expression of S1 and S2. M is pre-stained protein marker. lanes 1 and 3 are S0 and S1/S2 induced by 0.5 mmol·l^-1^ IPTG for 0 h. lanes 2 and 4 are S0 and S1/S2 induced by 0.5 mmol·l^-1^ IPTG for 16 h.

We performed PCR amplification using the synthesized *Hped* and *EbA309* genes as templates and expression primers to obtain the *Hped* gene with a size of 747 bp and the *EbA309* gene with a size of 819 bp (Fig. 2A and 2B). Then, they were ligated separately to the pET-28a expression vector. To identify whether the recombinant plasmids were successfully constructed, they were digested doubly with NcoI and XhoI. As shown in Fig. 2C and 2D, 5,369, 5,369-bp pET-28a vector, 747-bp *Hped* gene and 819-bp *EbA309* gene were observed. The constructed pET-28a-*Hped* and pET-28a-*EbA309* plasmids were introduced separately into *E. coli* BL21 (DE3), and the expression of *Hped* and *EbA309* genes was identified by SDS-PAGE electrophoresis after induction. The results demonstrated that the engineered *E. coli* strains used in subsequent biotransformation had been successfully constructed because the expression bands in S1 and S2 were comparable to the theoretical molecular weights of *Hped* (26.2 kDa, Fig. 2E) and *EbA309* proteins (27.6 kDa, Fig. 2F), respectively.

### Comparison of Biotransformation Activity between S1 and S2

**Fig. 3:**
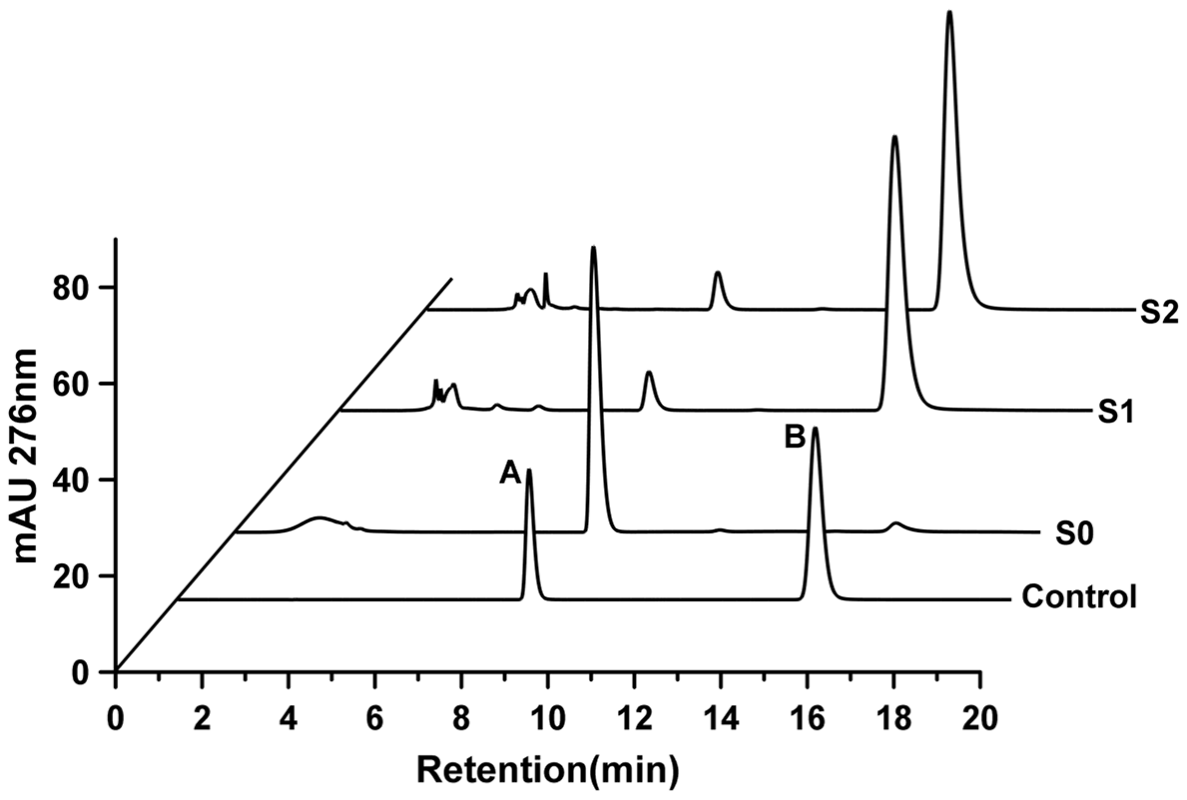
HPLC analysis. A: 1-(4-hydroxyphenol)-ethanol standard, B: ρ-hydroxyacetophenone standard, S0: *E. coli* containing pet-28a plasmid, S1: *E. coli* containing pET-28a-*Hped* plasmid, S2: *E. coli* containing pET-28a-*EbA309* plasmid.

**Fig. 4:**
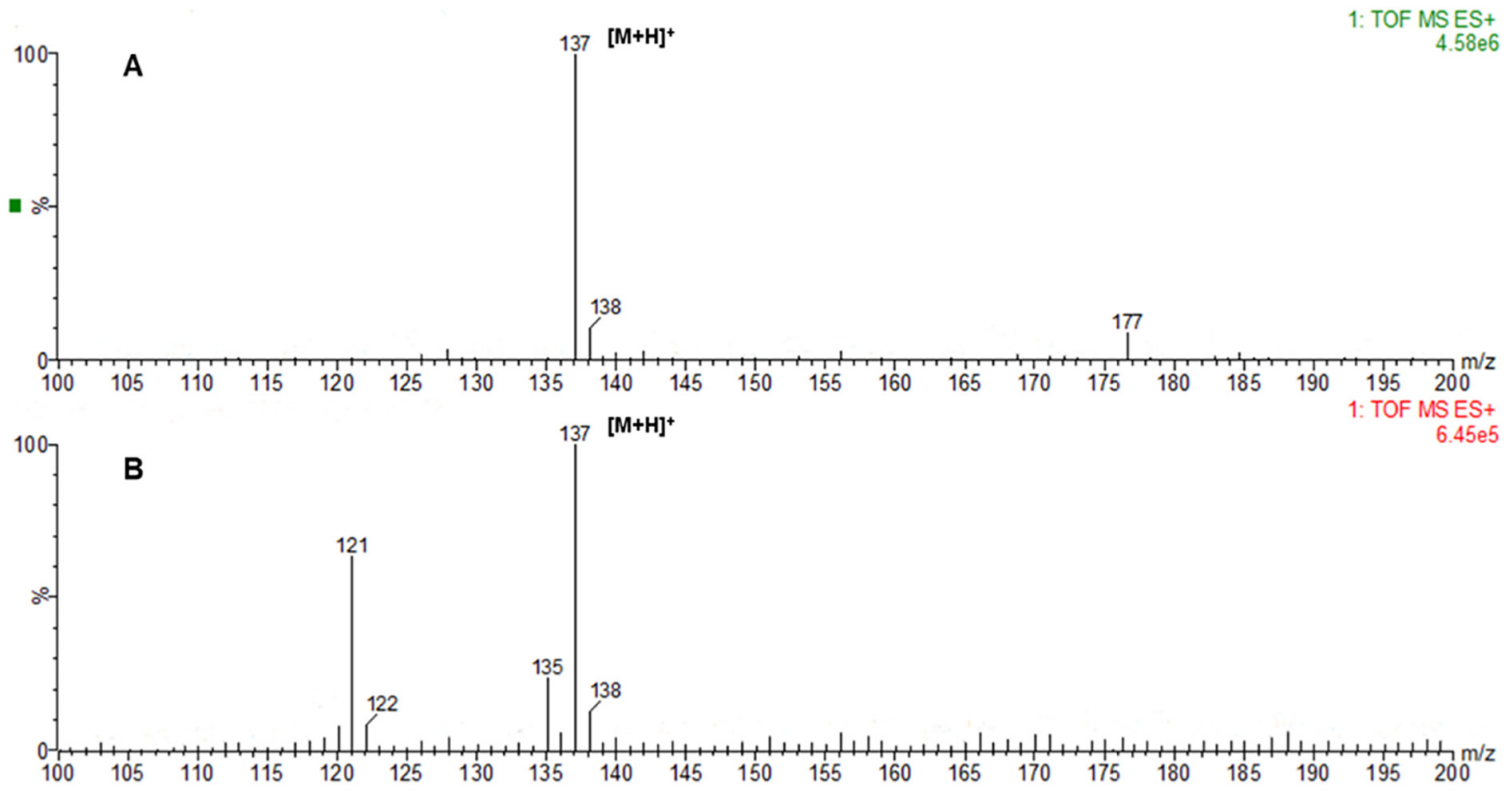
Mass spectrometry detection. (**A**) Standard mass spectra of ρ-HAP; (**B**) Mass spectra of the substances produced by the conversion of S1 and S2. 137 is the m/ z of ρ-HAP plus H^+^.

**Fig. 5:**
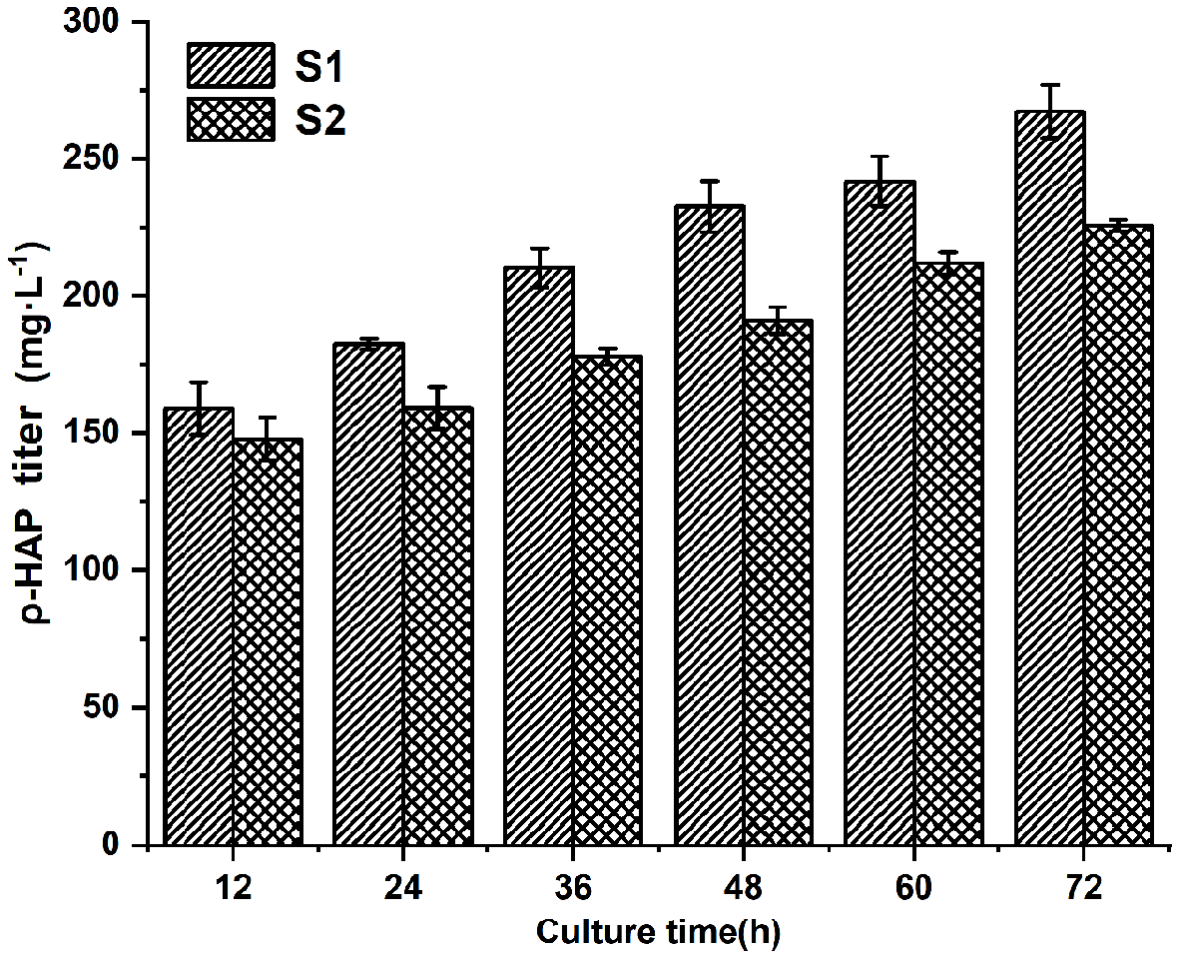
Comparison of biotransformation activity. All data points are reported as mean ± s.d. from three biologically independent samples.

Following induction, S0, S1, and S2 were transferred to an M9Y medium with 4 mmol/l 1-(4-hydroxyphenol)-ethanol for biotransformation. Then, 24 h later, HPLC was used to determine whether ρ-HAP was produced in the corresponding samples. As shown in Fig. 3, the S1 and S2 samples had chromatographic peaks with the same retention time as that of ρ-HAP, but the S0 sample did not. Further identification by mass spectrometry showed that S1 and S2 had chromatographic peaks with the same mass-to-charge ratio (m/z) to that of ρ-HAP (Fig. 4). This indicated that S1 and S2 could transform 1-(4-hydroxyphenol)-ethanol into ρ-HAP. Under the same transformation conditions, the abilities of S1 and S2 to convert 1-(4-hydroxyphenol)-ethanol to ρ-HAP were compared. As shown in Fig. 5, the amount of ρ-HAP produced by S1 was 158.97 ± 9.7 mg/l (conversion rate = 29.2%), 182.42 ± 1.9 mg/l (conversion rate = 33.5%), 210.23 ± 7.1 mg/l (conversion rate = 38.6%), 232.58± 9.3 mg/l (conversion rate = 42.7%), 241.74 ± 9.1 mg/l (conversion rate = 44.4%) and 267.19 ± 9.7 mg/l (conversion rate = 49.1%) at 12, 24, 36, 48, 60, and 72 h, respectively. The amount of ρ-HAP generated by S2 was 147.81 ± 7.8 mg/l (conversion rate = 27.1%), 159.06 ± 7.8 mg/l (conversion rate = 29.2%), 177.73 ± 3.1 mg/l (conversion rate = 32.6%), 191.01 ± 4.9 mg/l (conversion rate = 35.1%), 211.87 ± 4.1 mg/l (conversion rate = 38.9%) and 225.57 ± 2.0 mg/l (conversion rate = 41.4%) at 12, 24, 36, 48, 60, and 72 h, respectively. The results indicated that the ability of S1 to transform the substrate into the product was stronger than that of S2. Thus, S1 was chosen for further exploration of the optimal condition for subsequent biotransformation.

### Exploration of Biotransformation Conditions

**Fig. 6:**
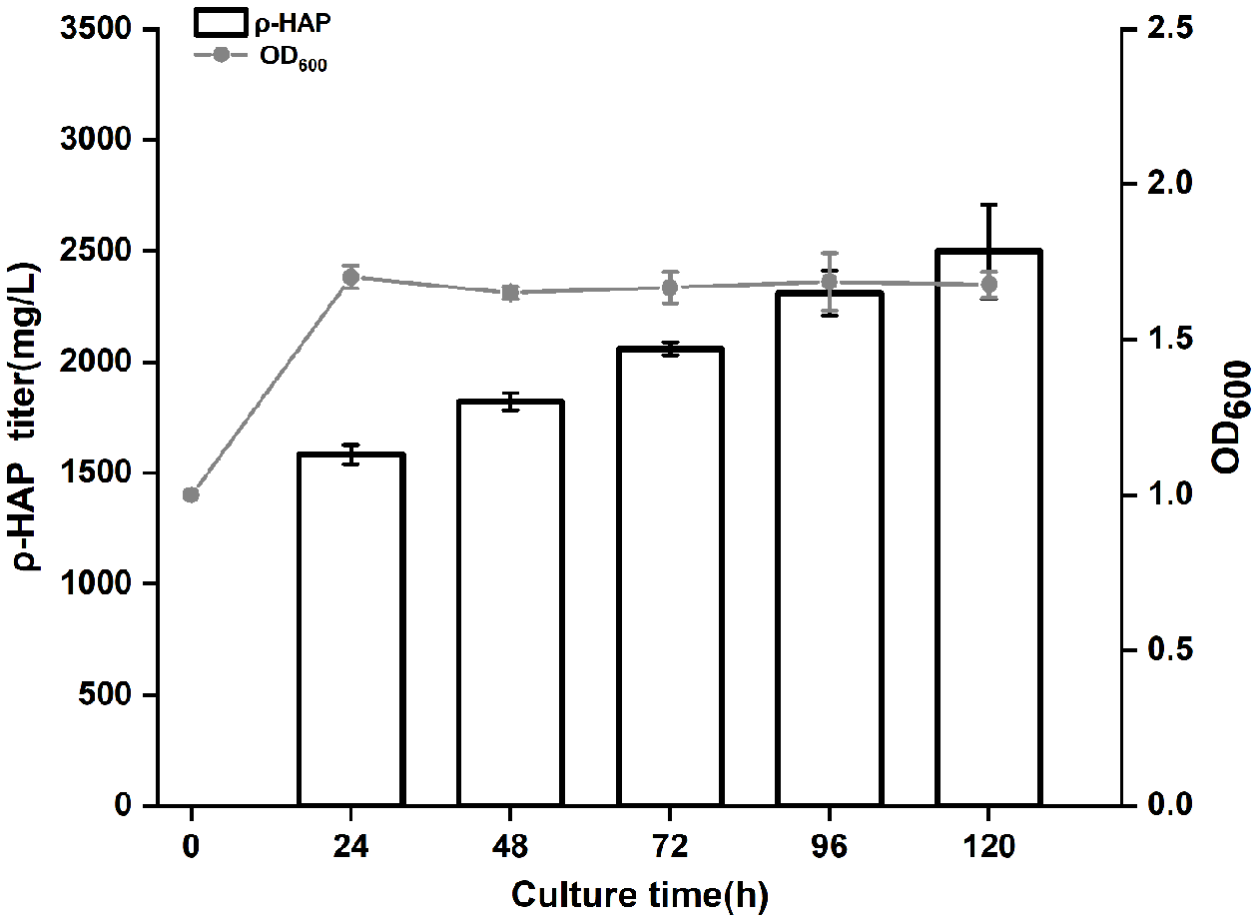
The effect of biotransformation time on the ρ-HAP concentration and cell growth. All data points are reported as mean ± s.d. from three biologically independent samples.

**Fig. 7:**
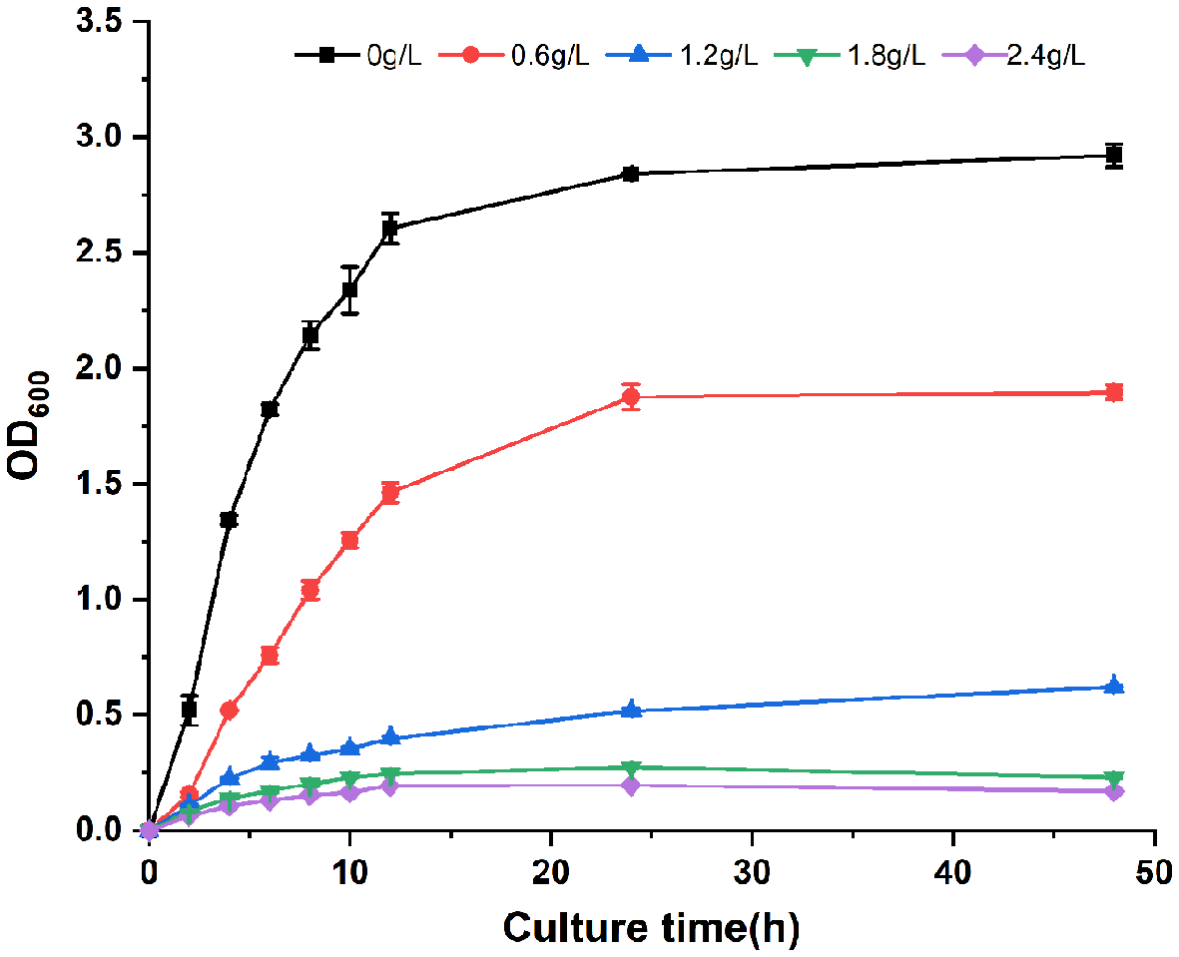
The inhibitory effect of ρ-HAP on *E. coli*. All data points are reported as mean ± s.d. from three biologically independent samples.

Four factors including substrate concentration, IPTG concentration, induction temperature and transformation temperature were selected as independent variables. The change of the ρ-HAP concentration was taken as a dependent variable. Orthogonal tests with 4 factors and 3 levels were conducted according to the independent and dependent variables. The results of the orthogonal tests and range analysis are shown in Table 4, and the results of variance analysis are shown in Table 5. Based on the R value in the range analysis and the F value in the variance analysis in Tables 4 and 5, it could be concluded that the primary and secondary order of various factors affecting the ρ-HAP concentration was D> B> C> A, namely, substrate concentration > IPTG concentration > induction temperature > transformation temperature. The analysis of the mean K showed that the best combination was A_2_ B_1_ C_2_ D_3_. Under the optimal condition, the effects of transformation time on the ρ-HAP concentration and cell growth were further explored. The results (Fig. 6) indicated that during the selected time period, the ρ-HAP concentration increased steadily. However, when the ρ-HAP concentration increased to 1583.19 ± 44.34 mg/l in 24 h, cell growth was inhibited and then it entered a plateau. To confirm the inhibitory effect of ρ-HAP on *E. coli*, we added ρ-HAP to the LB medium to form a concentration gradient from 0 to 2.4 g/l, and then we determined the OD_600_ values of S0 at different time points. As shown in Fig. 7, with the gradual increase of the ρ-HAP concentration, the OD_600_ value of S0 gradually decreased. Especially, when the final concentration was greater than 1.8 g/l, cell growth was almost completely inhibited.

## Discussion

Microbes are the most abundant and diverse organisms on Earth. Many useful metabolic pathways and related enzymes in their cells have been discovered and used to synthesize a variety of high value-added chemicals [14, 15]. The *Aromatoleum aromaticum* strain EbN1 was originally isolated as a bacterium for anaerobic degradation of ethylbenzene [16]. With the deepening of research, it was found that strain EbN1 could also utilize other aromatic compounds. As a result, EbN1 was used as the first model bacterium for anaerobic degradation of aromatic compounds, and its genome was sequenced and annotated [17]. According to the annotation results, more than ten enzyme genes associated with anaerobic degradation of aromatic compounds have been discovered, such as *Hped* and *EbA309* genes [18-21]. Although the aforementioned research contributed to the discovery of *Hped* and *EbA309* genes, it was not reported how to use them, especially their expression products, to catalyze the conversion of 1-(4-hydroxyphenol)-ethanol to ρ-HAP. Accordingly, our study focused on this issue.

As a model microorganism, *E. coli* was used to express *Hped* and *EbA309* genes for two main reasons. On the one hand, both *E. coli* and EbN1 belong to bacteria, so they have a similar intracellular environment, which is beneficial to the expression of active *Hped* and *EbA309* proteins in *E. coli*. On the other hand, *E. coli* has the advantages of complete genome information, simple molecular operation, short growth cycle, low requirement for culture conditions, and facile separation of transformed products [22], all of which are helpful in furthering genetic modification and industrial production. After we selected *E. coli*, the engineered bacteria containing *Hped* or *EbA309* genes were constructed by genetic engineering, and their abilities to produce ρ-HAP were compared. On this basis, orthogonal tests with four factors and three levels were performed on S1, with its stronger transformation ability, to determine the primary and secondary order and the optimal transformation condition. Among the transformation conditions, substrate concentration was the most crucial, because the presence or absence of the substrate directly affected the occurrence of enzymatic reactions, and its concentration also affected the product yield. The factor that ranked second was IPTG concentration. As an inducer, IPTG can promote the expression of genes with the increase of its concentration, but its toxicity against cells will also increase. Therefore, it was reasonable to fix the optimal level at 0.1 mmol/L in the orthogonal tests. It not only produced a favorable induction effect, but also did not affect cell growth. The factor that ranked third was induction temperature, which determined the velocity and quality of gene expression. When the induction temperature rises to within a certain range, the quantity of enzyme proteins increases as the expression rate of enzyme genes speeds up. However, when the temperature exceeds a certain threshold, enzyme proteins often form a large number of inclusions in cells since it is too late for them to fold. Conversely, when the induction temperature is lowered to within a given range, the expression rate of the enzyme genes will slow down to extend the time for the folding of enzyme proteins encoded by them. However, the number of enzyme proteins will decrease, which eventually leads to a reduction in the product yield. Consequently, 25°C was determined by the orthogonal tests as a moderate induction temperature. The factor that ranked fourth was transformation temperature, which affects the catalytic activity of the enzyme if it is too high or too low. For instance, Tataruch *et al*. reported that *Hped* proteins would be inactivated when the temperature range was 47.5~60°C [23]. Thus, it was in line with the theoretical prediction and research report that 35°C was the optimal temperature in the three-level orthogonal tests. In addition, in terms of the effects of transformation time on the ρ-HAP concentration and cell growth, the ρ-HAP concentration increased continuously during the selected transformation time period. However, when the concentration of ρ-HAP reached 1583.19±44.34 mg/L in 24 h, it slowly inhibited cell growth. Hence, it was necessary to further explore how to reduce the influence of high concentrations of ρ-HAP on cell growth and continuously improve the ability of engineered *E. coli* to produce ρ-HAP.

The *Hped* protein is an NAD^+^-dependent dehydrogenase, which transforms NAD^+^ into NADH+H^+^ after dehydrogenation of the substrate in the cell. This change caused by the *Hped* protein would seem to upset the redox balance in *E. coli*, but it doesn't happen in fact. The reason is that *E. coli* needs a lot of NADH+H^+^ to provide reducing power in various life activities, which leads to the enormous consumption of NADH+H^+^ and the rapid regeneration of NAD^+^. For instance, there is a flavin reductase (Fre) that can efficiently convert NADH+H^+^ to FADH_2_ in *E. coli* and plays an important role in maintaining the balance of NAD^+^/NADH+H^+^ [24]. In addition, the precursor of NAD^+^ is nicotinic acid, which can be synthesized from tryptophan in *E. coli* [25]. In this study, yeast extract rich in tryptophan was added to the M9Y culture medium, which was beneficial for supplementing NAD^+^ needed by *Hped* protein. In a recent study, Lu *et al*. established a biosynthetic pathway for the production of ρ-HAP from glucose [26]. They promoted the titer of ρ-HAP by regulating intracellular redox cofactors influencing *Hped* proteins. In our study, by optimizing the culture conditions that influenced *Hped* proteins, such as substrate concentration, IPTG concentration, and induction temperature, the titer of ρ-HAP produced by S1 was significantly enhanced. Although the methods used in the two studies are different, both of them can effectively improve the production of ρ-HAP. Thus, they can be combined to produce ρ-HAP in future studies.

Chemical synthesis is the main method used for obtaining ρ-HAP at present, and the ρ-HAP yield can reach 70~90% [11]. Fries rearrangement is a core reaction in the process of chemical synthesis and requires not only high temperature and high pressure, but also the participation of catalysts, such as Lewis acids, BrÖnsted acid, or strong protoic acid [27]. However, these catalysts are strongly corrosive, have poor selectivity, and are environment unfriendly. Compared with chemical synthesis, biotransformation of engineered *E. coli* is a novel attempt to synthesize ρ-HAP with some exclusive advantages. For example, the biotransformation conditions used in this method are normal temperature and normal pressure, and various reagents used for *E. coli* culturing are not only cheap but also eco-friendly. However, biotransformation also has some shortcomings, such as low conversion rate, costly substrates, and complicated operation of shaking bacteria. Therefore, it is necessary to solve the existing problems in future research, so that the synthesis of ρ-HAP by biotransformation can be applied on a large scale.

## Conclusion

In this study, an engineered *E. coli* strain (S1) containing the *Hped* gene and another engineered *E. coli* strain (S2) containing the *EbA309* gene were successfully constructed. The S1 strain with higher activity was screened out. The ability of S1 to transform 1-(4-hydroxyphenol)-ethanol into ρ-HAP was influenced by substrate concentration, IPTG concentration, induction temperature, and transformation temperature. The order of the four factors was D>B>C>A, and the optimal condition was A_2_B_1_C_2_D_3_. On this basis, the effects of transformation time on the ρ-HAP concentration and cell growth were further studied. The results showed that although the ρ-HAP concentration gradually increased with the continuous extension of transformation time, cell growth was inhibited when the ρ-HAP concentration increased to 1583.19±44.34 mg/L^-1^ in 24 h. In conclusion, this study has achieved the objective of using engineered *E. coli* to convert 1-(4-hydroxyphenol)-ethanol to ρ-HAP. The findings lay a preliminary foundation for further improvement and development of the biosynthesis method of ρ-HAP in the future.

## Figures and Tables

**Table 1:** Strains and plasmids used in this study.

Name	Description	Source
Strains		
BL21(DE3)	F-, ompT, hsdS(rBB-mB-), gal, dcm (DE3)	Novagen
S0	BL21(DE3) harboring plasmid pet-28a	This study
S1	BL21(DE3) harboring plasmid pet-28a-*hped*	This study
S2	BL21(DE3) harboring plasmid pet-28a-*EbA309*	This study
Plasmids		
pet-28a	Expression vector, Kana^R^	Novagen
pet-28a-*hped*	pet-28a containing *hped*, Kana^R^	This study
pet-28a-*EbA309*	pet-28a containing *EbA309*, Kana^R^	This study

**Table 2:** Primer sequences.

Primer name	Primer sequence (5’→3’)
P-*Hped*-F	CATGCCATGGGAATGCTGCTGGAAGGC
P-*Hped*-R	CCGCTCGAGTTAGCGCGCTAAGTAGCC
P-*EbA309*-F	CATGCCATGGGCATGAAGCAGAATCTG
P-*EbA309*-R	CCGCTCGAGTCAACTAATGGTCATACC

Underlines represent restriction site, CCATGG is NcoI, and CTCGAG is XhoI.

**Table 3:** Factors and levels of orthogonal tests.

Levels	Factors			
	Substrate concentration / mmol·l^-1^	IPTG concentration /mmol·l^-1^	Induction temperature /°C	Biotransformation temperature /°C
1	0.4	0.1	15	25
2	4	0.5	25	35
3	40	1.0	35	45

**Table 4:** Results of the orthogonal tests.

Test number	Factors				Concentration / mg·l^-1^
	A Biotransformation temperature	B IPTG concentration	C Induction temperature	D Substrate concentration	
1	1	1	1	1	12.71
2	1	2	2	2	175.62
3	1	3	3	3	442.91
4	2	1	2	3	679.34
5	2	2	3	1	50.37
6	2	3	1	2	268.65
7	3	1	3	2	366.22
8	3	2	1	3	155.20
9	3	3	2	1	50.37
K_1_	210.41	352.76	145.52	37.82	
K_2_	332.79	127.06	301.78	270.16	
K_3_	190.60	253.98	286.50	425.82	
R value	142.19	225.69	156.26	388.00	
Primary and secondary order	D>B>C>A				
Optimal level	A_2_	B_1_	C_2_	D_3_	

**Table 5:** Results of variance analysis.

Source of variation	Type III sum of squares	Free degree	Mean square	F value	*p* value	Significance
Modified model	1157002.29	8	144625.286	15.961	0.000	**
Intercept	1615363.545	1	1615363.545	178.274	0.000	**
Biotransformation temperature	230399.040	2	115199.520	12.714	0.000	**
IPTG concentration	106759.297	2	53379.648	5.891	0.011	**
Induction temperature	133576.903	2	66788.452	7.371	0.005	**
Substrate concentration	686267.048	2	343133.524	37.869	0.000	**
Error	163100.076	18	9061.115			
Total	2935465.909	27				

*Indicates a significant factor effect (*p* < 0.05); **Indicates a highly significant factor effect (*p* < 0.01).
